# Associations of Illness Perception, Resignation Coping, and Social Support With Self‐Regulatory Fatigue in Patients With Type 2 Diabetes: A Cross‐Sectional Study

**DOI:** 10.1002/nop2.70781

**Published:** 2026-08-30

**Authors:** Qian Liu, Yijing Zhou, Bing Li, Qiuru Zhuang, Zhier Lin, Zhenrong Zhang, Xiaoying Yan, Kun Zeng, Weiyan Yuan

**Affiliations:** ^1^ School of Nursing Guangdong Pharmaceutical University Guangzhou China; ^2^ Science and Education Section Dongguan Eighth People's Hospital (Dongguan Children's Hospital) Dongguan China

**Keywords:** coping style, illness perception, self‐regulatory fatigue, social support, Type 2 diabetes

## Abstract

**Aims:**

To examine the associations among illness perception, resignation coping, social support, and self‐regulatory fatigue (SRF) in patients with Type 2 diabetes mellitus. The study specifically explores whether resignation coping and social support exhibit indirect associations with SRF in the context of illness perception.

**Design:**

A cross‐sectional study.

**Methods:**

From November 2024 to October 2025, a convenience sample of 302 adult patients with T2DM was recruited from a tertiary general hospital in China. Participants completed validated instruments with established psychometric properties, namely the Brief Illness Perception Questionnaire, the Medical Coping Modes Questionnaire, the Perceived Social Support Scale, and the Self‐Regulatory Fatigue Scale. Statistical analyses included Spearman correlation and serial mediation analysis using the PROCESS Macro (Model 6) with bias‐corrected bootstrapping.

**Results:**

SRF was positively correlated with negative illness perception (*r* = 0.579, *p* < 0.01) and resignation coping (*r* = 0.612, *p* < 0.01), and negatively correlated with social support (*r* = −0.598, *p* < 0.01). Serial mediation analysis revealed that illness perception was associated with SRF through indirect pathways involving resignation coping and social support. Resignation coping and social support mediated the association between illness perception and self‐regulatory fatigue, both individually and sequentially.

**Conclusions:**

Negative illness perception correlates with higher SRF. This observed correlation is additionally linked to indirect pathways involving resignation coping and lower social support. Collectively, these findings highlight a pattern of interrelated cognitive (illness perception), behavioural (resignation coping), and resource (social support) factors that are associated with self‐regulatory fatigue in this cross‐sectional study.

**Implications for the Profession:**

The findings offer a clear, evidence‐based framework for nursing practice. They highlight the potential value of integrated assessment and intervention that simultaneously addresses patients' illness beliefs, maladaptive coping strategies, and social support systems, which are associated with lower SRF and better diabetes self‐management.

**Reporting Method:**

This study adheres to the Strengthening the Reporting of Observational Studies in Epidemiology (STROBE) guidelines for cross‐sectional studies.

**Patient or Public Contribution:**

Patients participated solely as research participants by providing survey data. They were not involved in the study design, implementation, data analysis, interpretation, or manuscript preparation. All patients provided written informed consent prior to questionnaire completion.

## 1 Introduction

The global prevalence of diabetes continues to rise, posing a major public health challenge (GBD 2021 Diabetes Collaborators 2023). China bears the heaviest diabetes burden worldwide, with approximately 140 million adults affected by diabetes, over 90% of whom have Type 2 diabetes mellitus (T2DM) (Deng et al. 2024; Liu et al. 2025; Li et al. 2021). The chronicity of T2DM, along with its high risk for debilitating complications, severely impairs patients' quality of life (Murugadoss et al. 2026). Modern diabetes management emphasizes patient self‐management as the cornerstone of effective care. However, a significant gap persists between knowledge and sustained action, with only about 40% of patients maintaining long‐term adherence to recommended regimens (Highton et al. 2026; Archer and Llahana 2026; Chan et al. 2024). This pervasive “intention‐behaviour gap” highlights the need for research to identify specific psychological factors associated with the execution of self‐care behaviours, moving beyond explanations based solely on knowledge or motivation.

This study focuses on SRF as a key factor associated with behavioural execution failure. The theoretical foundation of SRF lies in the Strength Model of Self‐regulation, which posits that the psychological resources required for active self‐control are finite (Evans et al. 2016; Nes et al. 2013). For patients with T2DM, the continuous demands of disease management—including diet, medication, physical activity, and blood glucose monitoring—progressively correlate with the depletion of these resources. Compared with broader constructs such as diabetes distress (emotional suffering), self‐efficacy (belief in capability), or general burnout, SRF provides a more precise account of the “inability to execute” phase of self‐management. Importantly, SRF has been shown to correlate with adverse health outcomes, including poor glycemic control (elevated glycated haemoglobin) and diminished quality of life, underscoring its significant clinical and public health significance (Wang et al. 2023, 2018; Kuo et al. 2023).

The emergence of SRF is related to upstream cognitive and behavioural processes. Illness perception encompasses patients' cognitive and emotional representations of their identity, aetiology, timeline, consequences, and controllability, constituting a core primary appraisal process within the Transactional Model of stress and coping (Lu et al. 2025; Abdollahi et al. 2022). Evidence indicates that negative illness perception correlates with psychological distress, feelings of helplessness, and depletion of psychological resources, factors which may, in turn, be associated with heightened SRF (Zhou et al. 2023; Hoogendoorn et al. 2024).

In the context of chronic illness‐related stress, coping style and social support are closely related to an individual's vulnerability to psychological exhaustion. Adaptive coping is associated with better stress management and preservation of psychological resources, whereas maladaptive coping strategies such as resignation are associated with greater negative affect and SRF (Kurniyawan et al. 2022; Hapunda 2022). Resignation coping, characterized by passive acceptance and relinquishment of control, is associated with feelings of powerlessness. Concurrently, social support from family, friends, and healthcare providers serves as a vital external buffer that is associated with stress mitigation and adaptation (Parviniannasab et al. 2024). Multiple studies have demonstrated that higher levels of social support are associated with lower psychological burden and reduced SRF in T2DM patients (Wang et al. 2023; Wang, Peng, et al. 2025; Huang et al. 2024).

Nevertheless, the serial interrelationships among these variables remain unclear, and findings from previous studies have been inconsistent (Knowles et al. 2020; Chen, Maimaitituerxun, et al. 2024; Zhang et al. 2023). According to the Stress and Coping Theory (Aneshensel and Mitchell 2014), an individual's cognitive appraisal of illness is associated with subsequent coping responses, which may, in turn, be associated with perceptions of external support resources. Integrating the Common‐Sense Model (CSM) (Leventhal et al. 2016), illness perception can be conceptualized as a theoretically antecedent cognitive‐emotional representation that may be associated with resignation coping and social support in relation to SRF. Accordingly, this study proposes a theory‐driven serial association model in which negative illness perception is associated with greater use of resignation coping, which in turn is associated with lower social support, and this pattern ultimately is associated with higher levels of SRF.

The variable sequence proposed in this study is grounded in theory‐driven hypotheses derived from the Stress and Coping Theory and the CSM (Aneshensel and Mitchell 2014; Leventhal et al. 2016). It does not represent a temporally verified causal order, given the cross‐sectional nature of the data. Therefore, this study examines a theory‐informed directional serial association model: “Illness Perception → Resignation Coping → Social Support → SRF”. This investigation aims to enrich understanding of the psychosocial factors associated with self‐regulatory fatigue in patients with T2DM and to provide a basis for developing integrated nursing interventions targeting illness perception, resignation coping, and social support. Specifically, the CSM posits illness perception as the antecedent cognitive representation; Stress and Coping Theory explains the sequential appraisal‐coping‐resource pathway; and the Strength Model of Self‐Regulation frames self‐regulatory fatigue as the outcome of self‐regulatory resource depletion. The study hypotheses are as follows:

*Resignation coping shows an indirect association between illness perception and SRF*.

*Social support shows an indirect association between illness perception and SRF*.

*Illness perception is indirectly associated with SRF through a sequential pathway of increased resignation coping and reduced social support*.

*Illness perception is positively associated with SRF*.

## 2 The Study

### 2.1 Aims and Objectives

This study aims to examine a theory‐driven serial multiple indirect association model, investigating the extent to which resignation coping and social support exhibit indirect associations between illness perception and SRF in Chinese adults with T2DM.

### 2.2 Technical Terms to Describe Aims

Serial mediation analysis: psychosocial correlates: self‐regulatory resource depletion.

### 2.3 Primary, Secondary Objectives

The primary objective was to test the hypothesized serial mediation model (Illness Perception → Resignation Coping → Social Support → SRF). Secondary objectives included examining the direct associations between all key variables.

## 3 Methods/Methodology

### 3.1 Design

A cross‐sectional study design was employed.

### 3.2 Valid and Reliable Instruments

#### 3.2.1 Demographic and Clinical Characteristics

A self‐designed questionnaire collected information on demographics (e.g., age, gender, education, income) and clinical characteristics (e.g., diabetes duration, comorbidities, treatment regimen, latest fasting blood glucose).

#### 3.2.2 Brief Illness Perception Questionnaire (BIPQ)

The BIPQ (Broadbent et al. 2006) was used in its validated Chinese version (Mei et al. 2015), which has demonstrated good reliability in Chinese breast cancer patients (Cronbach's *α* = 0.77). Eight scorable items cover cognitive representations (5 items), emotional representations (2 items), and illness comprehensibility (1 item). Each item is rated from 0 to 10, with three items reverse‐scored. Items 3, 4, and 7 are reverse‐scored. Total scores range from 0 to 80, with higher scores reflecting more negative perceptions. Cronbach's *α* for this study was 0.786.

#### 3.2.3 Medical Coping Modes Questionnaire (MCMQ)

The Chinese version of the MCMQ (Shen and Jiang 2000), based on the original by Feifel et al. (1987), was used to assess coping styles. The validation study in Chinese patients with chronic illnesses reported subscale Cronbach's *α* coefficients of 0.69, 0.60, and 0.76. The 20‐item instrument includes three subscales: Confrontation (8 items), Avoidance (7 items), and Resignation (5 items). Items are rated on a 4‐point frequency scale (1 = “never” to 4 = “very often”). Items 1, 4, 9, 10, 12, 13, 18, and 19 are reverse‐scored. Higher subscale scores indicate greater use of that coping style. In this study, Cronbach's *α* coefficients were 0.782 for Confrontation, 0.654 for Avoidance, and 0.842 for Resignation.

#### 3.2.4 Perceived Social Support Scale (PSSS)

The PSSS, originally developed by Zimet et al. (1990), was administered in its Chinese version, which has established psychometric properties and demonstrated good reliability (Cronbach's *α* = 0.84) in Chinese populations (Chen et al. 2016). The 12‐item scale measures support from family, friends, and significant others (4 items each). Responses are recorded on a 7‐point Likert scale (1 = “very strongly disagree” to 7 = “very strongly agree”). Total scores range from 12 to 84. Higher scores reflect greater perceived social support. In this study, Cronbach's *α* was 0.969.

#### 3.2.5 Self‐Regulatory Fatigue Scale (SRFS)

The SRFS, originally developed by Nes et al. (2017), was assessed using its Chinese version, which was validated by Wang et al. (2015), in a sample of Chinese adults (Cronbach's *α* = 0.84). The 16‐item scale comprises three subscales: Cognitive Control (6 items), Emotional Control (5 items), and Behavioural Control (5 items). Items are rated on a 5‐point Likert scale from 1 (“strongly disagree”) to 5 (“strongly agree”), with five items reverse‐scored. Items 1, 2, 5, 9, and 14 are reverse‐scored. Total scores range from 16 to 80, with higher scores indicating more severe SRF. In the present sample, Cronbach's *α* was 0.870.

### 3.3 Sampling and Recruitment

Participants were recruited using convenience sampling from a tertiary general hospital in China.

### 3.4 Sample Size and Statistical Power

Sample size was calculated using G*Power 3.1.9.7. For multiple regression with an effect size *f* ^2^ = 0.15, *α* = 0.05, power = 0.80, and 6 covariates plus the primary predictors, a minimum of 118 participants was required. Allowing for a 20% invalid response rate, at least 148 participants were planned. Additionally, methodological literature on indirect association analysis was consulted. Prior research indicates that for serial indirect association models using bootstrap testing with moderate pathway effect sizes, a sample size exceeding 200 is generally adequate (Sim et al. 2022). The regression‐based sample size calculation provides a conservative estimate for the mediation model; the final sample of 302 exceeds this minimum recommendation, indicating that the primary mediation analysis is adequately powered.

### 3.5 Quality Appraisal

First, common method bias was assessed using Harman's single‐factor test; the first common factor accounted for 32.21% of the variance, below the 40% threshold. A supplementary analysis using the common latent factor method within confirmatory factor analysis was performed. After introducing a common latent factor, the largest absolute change in standardized factor loadings was 0.106, well below the commonly used 0.20 threshold. Furthermore, multicollinearity diagnostics for all predictors included in the regression models yielded variance inflation factor (VIF) values consistently below 2.00. Collectively, these findings indicate that the data were not compromised by serious common method bias or multicollinearity concerns.

### 3.6 Data Abstraction

The target population comprised adult Chinese patients with T2DM. A total of 302 eligible participants were included in the final analysis.

### 3.7 Inclusion and/or Exclusion Criteria

Inclusion criteria: (1) Confirmed diagnosis of T2DM for ≥ 6 months; (2) Age ≥ 18 years; (3) Ability to complete questionnaires independently; (4) Provision of written consent. Exclusion criteria: (1) Severe psychiatric or cognitive impairment; (2) Acute complications or critical illness.

### 3.8 Data Sources/Collection

From November 2024 to October 2025, participants were recruited via convenience sampling from the inpatient endocrinology department and diabetes specialist outpatient clinic of a tertiary general hospital in China. To minimize bias, the survey was conducted anonymously, and no personally identifiable information was collected. Prior to the survey, participants were informed that the research was for scientific purposes only, that there were no right or wrong answers, and that their responses would not affect their clinical care. All investigators received standardized training before formal data collection and used uniform instructions to explain the study purpose and completion requirements. After providing written informed consent, participants independently completed paper‐based questionnaires; investigators provided clarification of item meanings only when necessary and did not guide responses. A total of 320 questionnaires were collected. Following verification for completeness and response patterns, 18 questionnaires with substantial missing data on key variables or discernible patterned responses were excluded, resulting in a final analytic sample of 302 valid questionnaires.

### 3.9 Data Analysis

Data were analysed using IBM SPSS 26.0 and AMOS 24.0. Descriptive statistics characterized the sample. Normality tests indicated that the scale variables were not normally distributed; therefore, Spearman correlations examined associations among variables. Given that total scores of multi‐item scales can be treated as approximately continuous and that OLS regression with HC3 robust standard errors is robust to moderate non‐normality in samples of this size (*N* = 302) (Norman 2010; Lumley et al. 2002), parametric mediation analysis is justified. Accordingly, the serial indirect association model was tested using the PROCESS macro (Version 4.0, Model 6) with OLS regression and HC3 heteroscedasticity‐robust standard errors. The significance of indirect associations was determined using bias‐corrected bootstrapping with 5000 resamples; associations were considered statistically significant if the 95% confidence interval (CI) excluded zero. Six variables were controlled as covariates based on theoretical frameworks, previous literature, and empirical studies on psychosocial outcomes in diabetes, including gender, age, educational level, duration of diabetes, diabetic complications, and other chronic comorbidities (Wang et al. 2023; Tang and Gao 2020; Erdoğan Yüce and Yıldırım 2025). Given the cross‐sectional nature of the data, all reported pathways are statistical associations and do not imply causal ordering.

### 3.10 Ethical Considerations

The study was approved by the Ethics Committee of Dongguan Eighth People's Hospital (approval number: LL2024110804) and conducted in accordance with the Declaration of Helsinki. All participants were informed of the study purpose, the voluntary nature of participation, and their right to withdraw, and all provided written informed consent. The questionnaire was administered anonymously; no personally identifiable information was collected. All data were maintained securely and confidentially by the research team and used exclusively for research purposes.

## 4 Results

### 4.1 Participant Characteristics

The study included 302 T2DM patients (53.0% male; mean age = 58.76 ± 12.89 years). Most participants were married (79.1%), lived in town areas (58.9%), had a junior high school education or less (49.0%), and reported a monthly income ≤ 3000 RMB (42.1%). Regarding clinical features, 38.1% had diabetes for over 10 years, 22.8% had a family history of diabetes, and 54.6% had other chronic comorbidities. Oral hypoglycemic agents were the most common treatment (70.9%). Detailed characteristics are presented in Table 1.

**TABLE 1:** Descriptive statistics of study participants (*N* = 302).

Variable	Category	*N*	Percentage (%)
Gender	Male	160	53.0
	Female	142	47.0
Age	18–44	38	12.6
	45–59	118	39.1
	≥ 60	146	48.3
Medical payment type	Self‐financed	22	7.3
	Medical insurance	249	82.5
	Other	31	10.3
Educational level	Junior high school and below	148	49.0
	High school/Technical school	92	30.5
	College degree and above	62	20.5
Occupational status	Employed	157	52.0
	Retired	79	26.2
	Unemployed	66	21.9
Residence	Towns	178	58.9
	Rural	124	41.1
Marital status	Married	239	79.1
	Single	19	6.3
	Divorced	13	4.3
	Widowed	31	10.3
Personal monthly income	≤ 3000 yuan	127	42.1
	3001–5000 yuan	72	23.8
	5001–7000 yuan	59	19.5
	> 7000 yuan	44	14.6
Smoking status	No or quit	208	68.9
	Yes	94	31.1
Duration of diabetes mellitus	6 months ~ 5 years	113	37.4
	6 ~ 10 years	74	24.5
	> 10 years	115	38.1
Family history of diabetes	Yes	69	22.8
	No	233	77.2
Complication	Yes	90	29.8
	No	212	70.2
Other chronic comorbidities	Yes	165	54.6
	No	137	45.4
Current treatment programmes	Diet control alone	30	9.9
	Oral hypoglycemic agent	214	70.9
	Insulin	34	11.3
	Combined insulin and oral hypoglycemic agents	24	7.9
Latest fasting blood glucose	< 7.0 mmol/L	66	21.9
	≥ 7.0 mmol/L	236	78.1

### 4.2 Descriptive Statistics

As shown in Table 2, mean scores were 55.34 (±9.73) for SRF, 51.61 (±9.08) for illness perception, and 47.69 (±16.79) for social support. For coping styles, mean scores were 17.36 (±4.02) for confrontation, 15.33 (±3.22) for avoidance, and 12.41 (±3.44) for resignation.

**TABLE 2:** Descriptive statistics for variables (*N* = 302).

Variable (score)	Minimum	Maximum	Number of items	Score (*M* ± SD)	Average score of items (*M* ± SD)
Total score of self‐regulatory fatigue	20	76	16	55.34 ± 9.73	3.46 ± 0.61
Cognitive control	10	28	6	21.00 ± 3.33	3.50 ± 0.56
Emotion control	5	25	5	17.24 ± 3.68	3.45 ± 0.74
Behaviour control	5	25	5	17.10 ± 3.85	3.42 ± 0.77
Total score of illness perception	13	75	8	51.61 ± 9.08	6.45 ± 1.13
Cognitive representation	3	50	5	32.66 ± 6.66	6.53 ± 1.33
Emotional representation	4	20	2	15.28 ± 2.90	7.64 ± 1.45
Illness comprehensibility	0	8	1	3.67 ± 1.67	3.67 ± 1.67
Total score of social support	12	84	12	47.69 ± 16.79	3.97 ± 1.40
Family support	4	28	4	18.86 ± 5.29	4.72 ± 1.32
Friend support	4	28	4	14.04 ± 6.38	3.51 ± 1.59
Significant others support	4	28	4	14.78 ± 6.36	3.70 ± 1.59
Total score of coping style	25	60	20	45.10 ± 5.18	2.26 ± 0.26
Confrontation	9	30	8	17.36 ± 4.02	2.17 ± 0.50
Avoidance	7	25	7	15.33 ± 3.22	2.19 ± 0.46
Resignation	5	20	5	12.41 ± 3.44	2.48 ± 0.69

Abbreviations: *M*, mean; SD, standard deviation.

### 4.3 Correlation Analysis

As shown in Table 3, Spearman correlation analysis revealed significant correlations among the key variables. Specifically, SRF showed strong positive correlations with illness perception (*r* = 0.579, *p* < 0.01) and resignation coping (*r* = 0.612, *p* < 0.01), while exhibiting a strong negative correlation with social support (*r* = −0.598, *p* < 0.01). Furthermore, illness perception was strongly positively correlated with resignation coping (*r* = 0.630, *p* < 0.01) and moderately negatively correlated with social support (*r* = −0.419, *p* < 0.01).

**TABLE 3:** Correlations between illness perception, social support, resignation coping and self‐regulatory fatigue (*N* = 302).

	1	2	3	4	5	6
1. Self‐regulatory fatigue	1					
2. Illness Perception	0.579**	1				
3. Social Support	−0.598**	−0.419**	1			
4. Confrontation coping	−0.562**	−0.345**	0.649**	1		
5. Avoidance coping	−0.007	−0.065	−0.031	−0.019	1	
6. Resignation coping	0.612**	0.630**	−0.506**	−0.443**	0.046	1

*Note:* The avoidance coping subscale of the MCMQ demonstrated marginal internal consistency in this sample (Cronbach's *α* = 0.654); therefore, correlations involving this subscale should be interpreted with caution.

** *p* < 0.01 (two‐tailed).

### 4.4 Chain Mediation Analysis

**FIGURE 1:**
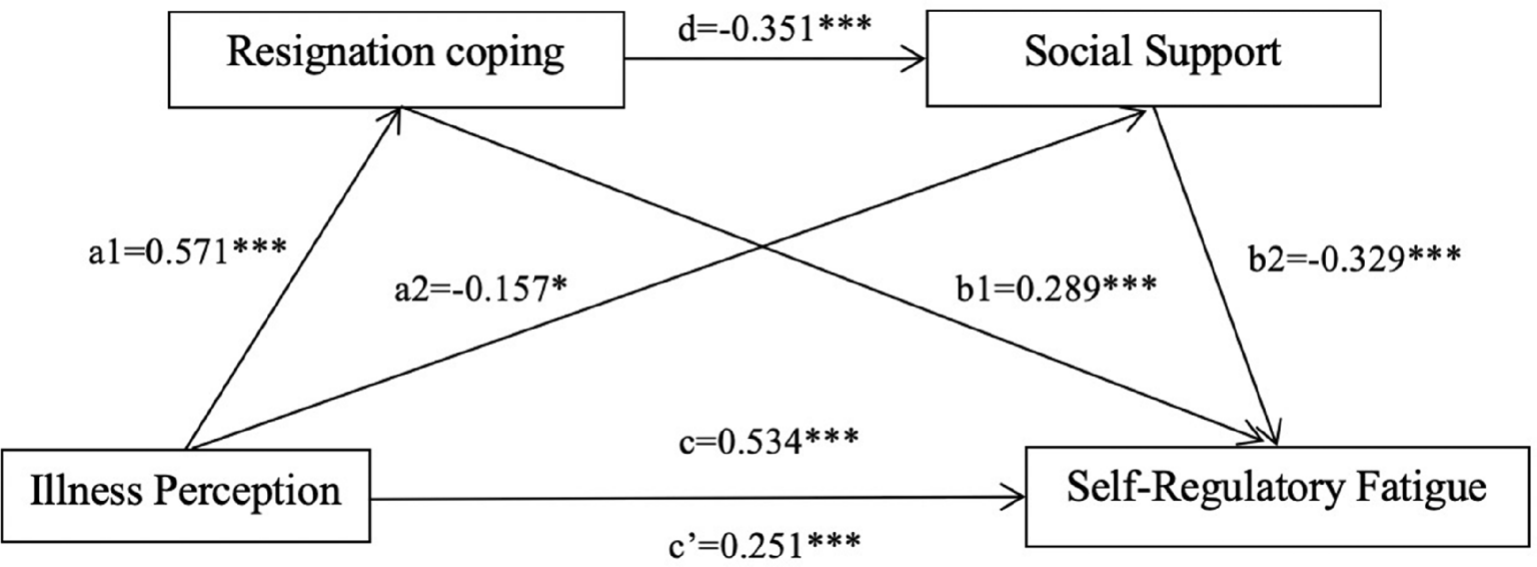
Serial mediation model for illness perception, social support, resignation coping and self‐regulatory fatigue. Path coefficients were shown in standardized regression coefficients. ***p < 0.001, *p < 0.05. All paths were adjusted for gender, age, educational level, diabetes duration, diabetes‐related complications, and other chronic comorbidities. *R* ^2^: Resignation coping = 0.467, Social Support = 0.311, Self‐Regulatory Fatigue = 0.565.

In the serial indirect association analysis, gender, age, educational level, diabetes duration, diabetes‐related complications, and other chronic comorbidities were included as covariates based on theoretical considerations, prior research, and clinical relevance (Table 4, Figure 1). Illness perception was positively associated with resignation coping (*β* = 0.571, *p* < 0.001). After controlling for resignation coping and the aforementioned covariates, illness perception was negatively associated with social support (*β* = −0.157, *p* = 0.039). Resignation coping was negatively associated with social support (*β* = −0.351, *p* < 0.001). The statistical direct effect of illness perception on SRF remained significant (*β* = 0.251, *p* < 0.001). Resignation coping was positively associated with SRF (*β* = 0.289, *p* < 0.001), while social support was negatively associated with SRF (*β* = −0.329, *p* < 0.001). The model accounted for 56.5% of the variance in SRF (*R* ^2^ = 0.565), indicating strong explanatory power.

**TABLE 4:** Regression analysis of variables in the chained multiple mediation model.

Outcome variables	Predictor variables	*B*	SE	*β*	*t*	*p*	*R* ^2^	*F*
Resignation coping	Illness perception	0.216	0.018	0.571	11.944	< 0.001	0.467	40.837***
Social support	Illness perception	−0.291	0.140	−0.157	−2.076	0.039	0.311	20.289***
	Resignation coping	−1.715	0.363	−0.351	−4.731	< 0.001		
Self‐regulatory fatigue	Illness perception	0.269	0.067	0.251	4.025	< 0.001	0.565	32.387***
	Resignation coping	0.818	0.192	0.289	4.258	< 0.001		
	Social support	−0.191	0.027	−0.329	−7.031	< 0.001		

*Note:* The reported *R* ^2^ and *F* for the social support outcome model correspond to the full adjusted model including illness perception, resignation coping, and all covariates.

Abbreviations: *β*, standardized coefficient; SE, standard error.

*** *p* < 0.001.

To examine the consistency of the primary associative patterns across key patient subgroups, supplementary subgroup analyses stratified by age group, diabetes duration, and complication status were conducted. The serial mediation pattern was broadly consistent in direction across subgroups; however, the statistical significance of individual indirect pathways varied. For instance, the 95% CIs for Ind2 in the ≥ 60 years group and Ind1 in the 6–10 years diabetes duration group included zero. Given the limited subgroup sample sizes, these exploratory findings should be interpreted with caution. Detailed results are presented in Tables S1–S3.

To test the hypothesized serial indirect association model, bootstrap analysis (Table 5) was conducted. The results revealed a total effect of illness perception on SRF of 0.572, with a direct effect of 0.269 and a total indirect effect of 0.303 (52.95% of the total). Specific indirect paths were:

- Path 1: Illness Perception → Resignation Coping → SRF (*B* = 0.177, 30.90%).
- Path 2: Illness Perception → Social Support → SRF (*B* = 0.055, 9.69%).
- Path 3: Illness Perception → Resignation Coping → Social Support → SRF (*B* = 0.071, 12.36%).

**TABLE 5:** Effect estimates for the hypothesized model.

	Effect (*B*)	SE	95% CI	*β*	Effect proportion
Total effect	0.572	0.063	[0.448, 0.695]	0.534	100%
Direct effect	0.269	0.067	[0.138, 0.401]	0.251	47.05%
Total indirect effect	0.303	0.047	[0.212, 0.398]	0.283	52.95%
Ind1	0.177	0.043	[0.095, 0.265]	0.165	30.90%
Ind2	0.055	0.026	[< 0.001, 0.103]	0.052	9.69%
Ind3	0.071	0.020	[0.036, 0.115]	0.066	12.36%

*Note:* The 95% confidence intervals for indirect effects were estimated using bootstrap procedures. Ind1 Illness Perception → Resignation Coping → Self‐Regulatory Fatigue; Ind2 Illness Perception → Social Support → Self‐Regulatory Fatigue; Ind3 Illness Perception → Resignation Coping → Social Support → Self‐Regulatory Fatigue. Effect proportion is calculated as the ratio of the specific indirect effect to the total effect of illness perception on self‐regulatory fatigue, not the proportion of the total indirect effect.

Abbreviations: *β*, standardized coefficient; *B*, unstandardized effect; CI, confidence interval; SE, standard error.

All 95% confidence intervals for indirect associations excluded zero, indicating significant serial indirect association pathways. The lower bound of the 95% CI for Ind2 was 0.0001 but is presented as < 0.001 due to rounding.

## 5 Discussion

Guided by and extending the Stress and Coping Theory, and integrating insights from both the CSM and the Strength Model of self‐regulation, this study provides cross‐sectional evidence consistent with a theory‐driven serial association model. The findings suggest that illness perception, resignation coping, social support, and SRF are interrelated in patients with T2DM. The core finding is an association pattern aligned with a “cognitive–behavioural–resource” sequence: negative illness perception demonstrates both a direct association with SRF and indirect associations manifested through resignation coping and social support.

### 5.1 Normative Comparison of Sample Characteristics

The illness perception score in this sample was comparable to previous T2DM studies (Zhang, Sun, et al. 2024), reflecting strong perceptions of disease threat, consequences, and emotional impact. The SRF score (55.34 ± 9.73) was moderate‐to‐high and higher than that reported by Wang et al. (2023), suggesting more pronounced depletion of psychological resources from long‐term self‐management, possibly due to the higher proportion of middle‐aged/older adults, longer disease duration, and greater treatment burden. Social support (47.69 ± 16.79) was moderate but lower than that reported by Chen, Chen, et al. (2024), reflecting relatively insufficient available or perceived support. Regarding coping styles, as measured by the average item score, resignation coping scored the highest (2.48 ± 0.69), consistent with prior evidence of passive coping tendencies (Zhang, Xu, et al. 2024), suggesting a tendency toward passive acceptance and helplessness under the sustained demands of chronic disease management.

### 5.2 Direct Association Between Illness Perception and SRF

In support of H4, negative illness perception was significantly associated with SRF. This aligns with the Stress and Coping Theory: appraising illness as threatening and uncontrollable is associated with continuous depletion of psychological resources (Ngetich et al. 2022). Similar findings have been reported in chronic illness studies linking negative perceptions to fatigue and poor quality of life (Zeng et al. 2024; Bagheri et al. 2022; Li et al. 2026). Illness perception is a core cognitive driver of T2DM self‐management (Xiong et al. 2024). Observing this association in a Chinese population suggests cross‐cultural stability. These findings imply that nurses should routinely assess illness perceptions and consider using brief cognitive‐behavioural techniques to address maladaptive beliefs—a strategy that may be associated with reduced SRF.

### 5.3 Indirect Association Involving Resignation Coping

This study supports H1, showing a significant indirect association of resignation coping within the relationship between illness perception and SRF. This pathway is congruent with a core premise of the Stress and Coping Theory: according to the model, threatening appraisal may be related to passive and depleting coping responses, which are directly associated with adverse outcomes. Resignation coping, characterized by relinquishing effort, is associated with depleted emotional resources and impaired adaptive problem‐solving, factors that are further associated with SRF (Chen et al. 2025). Prior research supports these associations; for instance, resignation and avoidance coping are associated with psychological distress and poor glycemic control. Similar indirect associations have been documented in Type 1 diabetes (Almeida et al. 2020). Therefore, nursing interventions that incorporate targeted skills training, including problem‐solving and cognitive restructuring, may be helpful in reducing the propensity for resignation coping.

### 5.4 Indirect Association Involving Social Support

This study supports H2, showing a significant indirect association of social support within the relationship between illness perception and SRF. Negative illness perception is associated with lower social support, which in turn is associated with heightened SRF. This pattern aligns with the buffering role of social support as an external resource. Notably, resignation coping was significantly negatively associated with social support, indicating that passive coping may be related to a reduced capacity to seek or perceive support (Kong et al. 2019; Yan et al. 2025). Social support can be viewed as a dynamic resource that may be jointly related to negative cognitions and maladaptive behaviours (Regeer et al. 2026; Zhang, Sun, et al. 2024). These findings lend support to the Stress and Coping Theory, suggesting that maladaptive coping such as resignation is associated with a less favourable perception of the social environment, thus being associated with a resource‐depleting cycle.

### 5.5 Sequential Mediation: A Vicious Cycle

The most novel finding of this study is the support for H3, showing a significant serial indirect association along the pathway illness perception → resignation coping → social support → SRF. This finding provides empirical support for the Stress and Coping Theory. The model posits that a negative appraisal is associated with a maladaptive coping response (resignation), which in turn is associated with diminished access to critical external resources (social support), and this sequence is associated with heightened SRF. This serial pathway extends beyond single indirect associations to illuminate the dynamic interrelationships among internal and external factors. This suggests that unidimensional interventions may have limited impact. Consequently, high‐quality nursing care could benefit from an integrated strategy that simultaneously addresses illness beliefs, coping skills, and support systems to comprehensively engage with this pathway. This study provides a foundation for developing comprehensive chronic disease nursing interventions that span cognitive, behavioural, and social dimensions.

However, the directional associations examined in this study are theoretically derived. Given the cross‐sectional design, reverse or bidirectional associations cannot be excluded. We acknowledge the potential for dynamic interplay among self‐regulatory fatigue, illness perceptions, coping style, and social support (Wang, Li, et al. 2025; Lu et al. 2025). For example, high levels of self‐regulatory fatigue may be associated with diminished psychological resources for actively reframing illness perceptions, which could limit the use of positive reappraisal strategies and thus be related to more negative illness perceptions. Additionally, social withdrawal and passivity associated with fatigue may reduce active help‐seeking, potentially contributing to a gradual decline in both received and perceived social support. These two potential pathways remain speculative and require testing in future longitudinal studies.

### 5.6 Theoretical and Practical Implications

Theoretically, this study empirically examines a serial indirect association model, refining the application of the Stress and Coping Theory and elucidating a sequential associative pattern from cognitive appraisal → maladaptive coping → erosion of social resources → depletion of self‐regulation. This integrative perspective connects the CSM, Stress and Coping Theory, and the Strength Model of Self‐Regulation, providing a comprehensive account of the associative pattern process from illness perception to resource exhaustion. Notably, the *R* ^2^ of 0.565 in our model is comparable to or slightly higher than those reported in some previous psychosocial studies on diabetes (Fang et al. 2026; Zhang, Sun, et al. 2024). However, given the differences in outcome variables, populations, and study contexts, direct cross‐study comparisons should be interpreted with caution. The present findings indicate that the integrated framework of illness perception, resignation coping, and social support captures a substantial proportion of the variance in self‐regulatory fatigue. This level of explained variance indicates that these three dimensions together account for a substantial proportion of the core modifiable psychosocial vulnerability factors. Translating these findings into practice, we propose a screening prioritization: any warning sign in these dimensions should prompt consideration of a targeted nursing pathway.

It is important to note that the following intervention framework represents a preliminary, theory‐derived proposal based on cross‐sectional associations and requires rigorous testing in randomized controlled trials before any clinical implementation. With this caveat, the present findings may inform a pathway‐oriented nursing approach that could comprise three core components: (1) Screening: During hospital admission or outpatient follow‐up visits, the SRFS, the BIPQ, the resignation subscale of the MCMQ, and the PSSS are used to rapidly identify high‐risk patients. (2) Intervention: A short‐term nursing intervention combining cognitive restructuring and behavioural activation is delivered once weekly for 20–30 min over 6 weeks. It integrates motivational interviewing, family collaboration task sheets, daily behaviour logs, and problem‐solving training, specifically addressing potentially modifiable factors (such as catastrophic illness perceptions and reliance on resignation coping) that may be associated with reduced self‐regulatory fatigue (Diriba et al. 2023; Nouri and Madi 2026; Zhu et al. 2025). (3) Referral and evaluation: At weeks 3 and 6 of the intervention, the SRFS is administered for dynamic assessment. If the score reduction is less than 20%, a joint referral to a diabetes nurse educator and a psychological counsellor is initiated.

### 5.7 Strengths and Limitations of the Study

Strengths include the use of instruments with established psychometric properties, robust bootstrap statistical methods, and control for key confounding variables. The study provides preliminary evidence for understanding the serial correlations among psychosocial factors in this population. Limitations are: (1) The cross‐sectional design limits the ability to determine temporal relationships and causal pathways; (2) Sample representativeness is limited. Participants were recruited from a single tertiary hospital in an eastern coastal region of China and were predominantly middle‐aged and older adults, covered by urban medical insurance, and with lower educational attainment. Clinically, the sample was weighted toward patients in the middle‐to‐late disease stages with a higher burden of comorbidities and complications. Therefore, caution is warranted when generalizing the findings to younger patients, those managed in primary care settings, self‐paying patients, or those with early‐stage, milder disease; (3) The supplementary subgroup analyses were exploratory in nature; limited sample sizes within certain strata preclude robust between‐group inferences; (4) The internal consistency of the avoidance coping subscale was marginally acceptable. (5) Although statistical tests suggested no severe common method bias, all data were self‐reported at a single time point, and common method bias cannot be completely ruled out.

### 5.8 Suggestions for Further Research

First, future research should employ longitudinal or experimental designs to clarify temporal relationships and explore causal inferences. Second, it is recommended that objective physiological indicators (e.g., HbA1c) and multi‐source data collection (e.g., combining self‐reports with clinician assessments or family reports) be incorporated, and that multi‐center, large‐sample designs encompassing heterogeneous populations across different disease stages and clinical profiles be adopted, so as to enhance the generalizability and reliability of conclusions. Methodologically, qualitative approaches such as semi‐structured interviews could be introduced to explore patients' subjective experiences and contextual factors related to resignation coping and lack of social support. Network analysis could also be employed to precisely identify core intervention targets within the serial association model. Finally, based on the pathways observed in this study, integrated nursing intervention protocols that combine cognitive restructuring, coping skills training, and social support mobilization should be developed and tested.

## 6 Conclusion

This study suggests that negative illness perception is associated with higher self‐regulatory fatigue in T2DM patients. Moreover, this association is partially related to the serial indirect pathway involving resignation coping and diminished social support. The findings indicate that clinical practice may benefit from an integrated care model that simultaneously addresses cognitive, behavioural, and social resource factors. The development and testing of multi‐component nursing interventions are recommended to engage with this pathway, with the goal of alleviating self‐regulatory fatigue and improving diabetes self‐management outcomes.

## Author Contributions

**Qian Liu:** conceptualization, methodology, writing – original draft. **Yijing Zhou:** validation, data curation. **Bing Li:** investigation, software. **Qiuru Zhuang:** validation, formal analysis. **Zhier Lin:** validation, formal analysis. **Zhenrong Zhang:** visualization, formal analysis. **Xiaoying Yan:** supervision. **Kun Zeng:** resources. **Weiyan Yuan:** funding acquisition, project administration, writing – review and editing.

## Funding

This research was funded by the Innovation Projects with Distinctive Features for Regular Higher Education Institutions in Guangdong Province (Grant No. 2023KQNCX030) and the Guangdong Province College Student Innovation Training Program (Grant No. S202510573007).

## Ethics Statement

The study was approved by the Ethics Committee of Dongguan Eighth People's Hospital (Approval No. LL2024110804) and conducted in accordance with the Declaration of Helsinki.

## Consent

All participants provided written informed consent.

## Conflicts of Interest

The authors declare no conflicts of interest.

## Supporting information


**Table S1:** Stratified serial indirect effects by age group.
**Table S2:** Stratified serial indirect effects by diabetes duration.
**Table S3:** Stratified serial indirect effects by complication status.

## Data Availability

The datasets used and analysed during the current study are available from the corresponding author on reasonable request.
