# Supplementary material for: Associations of Illness Perception, Resignation Coping, and Social Support With Self‐Regulatory Fatigue in Patients With Type 2 Diabetes: A Cross‐Sectional Study
Source: Nurs Open. 2026 Aug 30;13(9):e70781. doi: 10.1002/nop2.70781 (PMC13526466; doi:10.1002/nop2.70781)
Supplement: Supplementary file 1 [file NOP2-13-e70781-s001.docx]

**Supplementary Material**

**Table S1. Stratified serial indirect effects by age group**

| **Effect** | **< 60 years (n=156)** | | **≥ 60 years (n=146)** | |
| --- | --- | --- | --- | --- |
|  | **Effect value (B)** | **95% CI** | **Effect value (B)** | **95% CI** |
| Total effect | 0.564 | [0.384, 0.743] | 0.553 | [0.365, 0.741] |
| Direct effect | 0.273 | [0.076, 0.471] | 0.225 | [0.064, 0.387] |
| Total indirect effect | 0.291 | [0.166, 0.427] | 0.328 | [0.188, 0.480] |
| Ind1: IP → RC → SRF | 0.166 | [0.061, 0.289] | 0.224 | [0.087, 0.366] |
| Ind2: IP → SS → SRF | 0.067 | [-0.005, 0.130] | 0.019 | [-0.045, 0.090] |
| Ind3: IP → RC → SS → SRF | 0.057 | [0.017, 0.114] | 0.084 | [0.030, 0.156] |

Note. B = unstandardized coefficient; CI = bias-corrected bootstrap confidence interval (5,000 resamples). IP = illness perception; RC = resignation coping; SS = social support; SRF = self-regulatory fatigue. Covariates: gender, educational level, diabetes duration, diabetes-related complications, and other chronic comorbidities.

**Table S2. Stratified serial indirect effects by diabetes duration**

| **Effect** | **6 months–5 years (n=113)** | | | **6–10 years (n=74)** | | **>10 years (n=115)** | |
| --- | --- | --- | --- | --- | --- | --- | --- |
|  | **Effect value (B)** | | **95% CI** | **Effect value (B)** | **95% CI** | **Effect value (B)** | **95% CI** |
| Total effect | 0.598 | [0.379, 0.817] | | 0.599 | [0.363, 0.835] | 0.457 | [0.219, 0.695] |
| Direct effect | 0.222 | [-0.059, 0.503] | | 0.290 | [-0.038, 0.617] | 0.194 | [0.010, 0.379] |
| Total indirect effect | 0.376 | [0.219, 0.587] | | 0.309 | [0.076, 0.546] | 0.262 | [0.100, 0.432] |
| Ind1: IP → RC → SRF | 0.277 | [0.123, 0.503] | | 0.105 | [-0.082, 0.298] | 0.176 | [0.049, 0.325] |
| Ind2: IP → SS → SRF | 0.045 | [-0.047, 0.120] | | 0.128 | [0.007, 0.266] | -0.014 | [-0.109, 0.064] |
| Ind3: IP → RC → SS → SRF | 0.055 | [0.009, 0.141] | | 0.077 | [-0.001, 0.180] | 0.101 | [0.038, 0.186] |

Note. B = unstandardized coefficient; CI = bias-corrected bootstrap confidence interval (5,000 resamples). IP = illness perception; RC = resignation coping; SS = social support; SRF = self-regulatory fatigue. Covariates: gender, age, educational level, diabetes-related complications, and other chronic comorbidities.

**Table S3. Stratified serial indirect effects by complication status**

| **Effect** | **With complications (n=90)** | | **Without complications (n=212)** | |
| --- | --- | --- | --- | --- |
|  | **Effect value (B)** | **95% CI** | **Effect value (B)** | **95% CI** |
| Total effect | 0.589 | [0.375, 0.804] | 0.566 | [0.409, 0.722] |
| Direct effect | 0.262 | [0.036, 0.487] | 0.271 | [0.107, 0.435] |
| Total indirect effect | 0.328 | [0.142, 0.519] | 0.294 | [0.180, 0.412] |
| Ind1: IP → RC → SRF | 0.139 | [-0.031, 0.304] | 0.188 | [0.089, 0.298] |
| Ind2: IP → SS → SRF | 0.112 | [0.019, 0.230] | 0.039 | [-0.030, 0.097] |
| Ind3: IP → RC → SS → SRF | 0.077 | [0.010, 0.182] | 0.068 | [0.029, 0.122] |

Note. B = unstandardized coefficient; CI = bias-corrected bootstrap confidence interval (5,000 resamples). IP = illness perception; RC = resignation coping; SS = social support; SRF = self-regulatory fatigue. Covariates: gender, age, educational level, diabetes duration, and other chronic comorbidities.
